# Impact of Oral Health Interventions on Sarcopenia and Frailty in Older Adults: A Systematic Review

**DOI:** 10.3390/jcm14061991

**Published:** 2025-03-15

**Authors:** Andrés Celis, Benjamín Cáceres, Bárbara Escobar, Pilar Barahona, Erik Dreyer, Fanny Petermann-Rocha

**Affiliations:** 1 Faculty of Dentistry, Universidad de los Andes, Santiago 7620086, Chile; 2 Faculty of Dentistry, Universidad de Chile, Santiago 8431640, Chile; benjamin.caceres.o@ug.uchile.cl (B.C.); barbaraescobar@ug.uchile.cl (B.E.); pbarahona@odontologia.uchile.cl (P.B.); erikmda@yahoo.com (E.D.); 3 Faculty of Medicine, Universidad Diego Portales, Santiago 8370068, Chile; fanny.petermann@udp.cl

**Keywords:** frailty, sarcopenia, oral health, older adults

## Abstract

**Background/Objectives:** Frailty and sarcopenia are geriatric syndromes associated with increased vulnerability to adverse health outcomes, including functional decline, disability, and mortality. Emerging evidence suggests that oral health interventions may play a role in mitigating these conditions. This systematic review aims to evaluate the impact of oral health interventions on frailty and sarcopenia in older adults. **Methods:** A systematic search was conducted in PubMed, Scopus, and SciELO databases for studies published up to December 2023. Inclusion criteria comprised experimental and quasi-experimental studies assessing dental interventions and their effects on frailty and sarcopenia in individuals aged 60 years and older. The primary outcomes included frailty index, grip strength, walking speed, and functional dentition. Study quality was assessed using GRADEpro. **Results:** Eight studies were included. Preventive oral hygiene interventions improved oral health but did not significantly impact frailty scores. Oral exercises significantly improved muscle strength and weight, leading to frailty score reductions (−1.1 points, 95% CI: −1.5 to −0.7, *p* < 0.01). Swallowing therapies were linked to increased grip strength (+1.8 kg, *p* = 0.03) and walking speed (+0.2 m/s, *p* = 0.04), with corresponding frailty index reductions (−0.8 points, 95% CI: −1.2 to −0.4, *p* = 0.01). The certainty of evidence ranged from very low to moderate. **Conclusions:** Oral health interventions, particularly oral exercises and swallowing therapies, show potential in reducing frailty and sarcopenia-related outcomes in older adults. However, methodological heterogeneity and low-certainty evidence highlight the need for high-quality, large-scale trials with standardized assessment measures to establish definitive clinical recommendations.

## 1. Introduction

Frailty and sarcopenia are increasingly recognized as critical geriatric syndromes that not only compromise the physical functionality and independence of older adults but also amplify the risk of adverse outcomes, including falls, hospitalization, institutionalization, and mortality [1,2]. Frailty represents a state of heightened vulnerability due to diminished physiological reserves, while sarcopenia—the progressive loss of skeletal muscle mass and strength—is a key component and driver of functional decline in this population [3,4]. These conditions often co-exist and are linked to significant societal and economic burdens, making their prevention and management a public health priority [5].

Oral health, an often-overlooked determinant of systemic health, plays a pivotal role in the onset and progression of frailty and sarcopenia. Conditions such as tooth loss, periodontal disease, and impaired masticatory function are not merely localized issues but have far-reaching consequences on nutritional status, systemic inflammation, and overall health [6,7,8]. The emerging concept of “oral frailty” underscores the cumulative impact of oral health deterioration on physical and cognitive decline, positioning it as a potential early marker for broader systemic vulnerabilities [9]. Despite the recognized importance of oral health, its integration into geriatric care frameworks remains fragmented and inconsistent, resulting in missed opportunities for early intervention in high-risk populations.

Current evidence highlights the bidirectional relationship between oral health and systemic conditions, suggesting that targeted oral health interventions could play a transformative role in mitigating frailty and sarcopenia [10,11,12]. Interventions such as the provision of dental caries treatment, prosthetic rehabilitation, oral health education, and periodontal therapy have shown promise in improving chewing efficiency, dietary intake, and overall quality of life in older adults [13,14]. However, existing studies often lack methodological rigor, longitudinal follow-up, and a focus on clinically meaningful outcomes, leaving critical gaps in understanding their effectiveness. Given that older adults frequently experience a decline in oral function concurrent with systemic deterioration, an integrated approach that addresses both local and systemic health issues is urgently needed.

The rationale behind exploring oral health interventions in the context of frailty and sarcopenia is multifaceted. Poor oral health can lead to inadequate mastication and subsequent malnutrition, a known risk factor for both muscle wasting and functional decline. In addition, chronic oral infections and periodontal disease may trigger systemic inflammatory responses that exacerbate muscle catabolism and overall physiological decline. As the population ages, there is an increasing need to develop intervention strategies that not only address oral health problems but also contribute to the prevention or mitigation of frailty and sarcopenia [15,16].

This systematic review aims to critically evaluate the impact of oral health interventions on frailty and sarcopenia in older adults. By synthesizing evidence from experimental and quasi-experimental studies, this review seeks to provide robust insights into the role of oral health management in improving clinical outcomes, enhancing functional capacity, and fostering holistic well-being in this vulnerable population. Moreover, it seeks to establish a foundation for integrating oral health into multidisciplinary geriatric care strategies, addressing an urgent need in the face of global population aging. In doing so, we also discuss the methodological limitations inherent in the current literature and propose recommendations for future research to optimize intervention protocols and outcome measures.

Understanding the interplay between oral health and systemic function is essential for developing comprehensive care strategies. As the evidence base grows, it is increasingly clear that maintaining optimal oral health may have cascading benefits for overall physical performance and quality of life. In the context of frailty and sarcopenia, where multiple factors converge to impair function, the potential for oral health interventions to serve as a modifiable risk factor is of significant clinical interest. The integration of oral health into broader healthcare strategies is not only timely but necessary to address the complex challenges faced by the aging population.

## 2. Materials and Methods

### 2.1. Study Design

The present study was conducted following the guidelines of the PRISMA (Preferred Reporting Items for Systematic Reviews) 2020 statement [17]. A detailed review protocol was developed prior to initiating the literature search to ensure a structured and reproducible methodology (PROSPERO registration CRD420251005540).

### 2.2. Search Strategy

A systematic literature search was conducted in PubMed, Scopus, and SciELO for studies published up to December 2023. The search strategy utilized a combination of Medical Subject Headings (MeSHs) and free-text keywords. Key terms included “Dental Care”, “Oral Health”, “Oral Health Intervention”, “Frailty”, “Sarcopenia”, and “Older Adults”. These terms were combined using Boolean operators (AND, OR) to capture all relevant studies. The search strategy was adapted for each database to account for differences in indexing and filtering. Only studies available in full text were considered, and no language restrictions were applied. A complete list of the search terms and the specific filters applied for each database is provided in Appendix A.

### 2.3. Inclusion and Exclusion Criteria

Studies were included in this review if they met the following criteria: (1) they were designed as experimental or quasi-experimental studies; (2) they evaluated participants aged 60 years or older; (3) the interventions focused on oral healthcare, such as prosthetic rehabilitation, oral health education, oral exercises, or swallowing therapies; and (4) the outcomes measured included indicators of frailty (e.g., Fried Frailty Index, Kihon Checklist) and/or sarcopenia (e.g., grip strength, walking speed, muscle mass assessments). Studies were excluded if they were observational without an intervention component, lacked sufficient methodological detail or full-text availability, or if the populations studied had medical conditions unrelated to frailty or sarcopenia that might confound the results.

### 2.4. Study Selection and Data Extraction

An initial search of the records was conducted by one researcher (A.C.), who also removed duplicate entries. Two reviewers (B.C. and B.E.) independently screened the titles and abstracts to determine eligibility. Following this screening, full-text articles were retrieved for studies deemed potentially eligible. Each article was then assessed independently by the two reviewers according to the inclusion and exclusion criteria. Any discrepancies that arose during the screening process were resolved through discussion or by consulting a third reviewer (A.C.). For each study included in the review, data were extracted regarding the study characteristics (such as author, year, country, and sample size), participant demographics, a detailed description of the oral health intervention, the primary and secondary outcomes measured, and the duration of follow-up. This systematic extraction of data ensured that all relevant information was captured for the subsequent narrative synthesis.

### 2.5. Quality Assessment

The quality of the included studies was evaluated using GRADEpro software (https://www.gradepro.org accessed on 13 October 2024) to assess the certainty of evidence for each outcome. Factors considered in this evaluation included the risk of bias, consistency of results, directness of evidence, imprecision, and potential publication bias. Each study’s methodological rigor was scrutinized, with particular attention given to aspects such as sample size, blinding procedures, and the use of control groups. Although many studies faced limitations such as small sample sizes and a lack of blinding, the quality assessment provided a balanced view of the strengths and weaknesses inherent in the current evidence base.

### 2.6. Data Synthesis

Due to heterogeneity in study designs, intervention protocols, and outcome measures, a meta-analysis was not performed. Instead, a narrative synthesis was conducted to summarize and integrate the findings across studies. This approach allowed for a comprehensive discussion of the effects of various oral health interventions on both frailty and sarcopenia, highlighting both the consistent trends and the methodological differences among the studies.

## 3. Results

### 3.1. Study Identification and Selection

**Figure 1:**
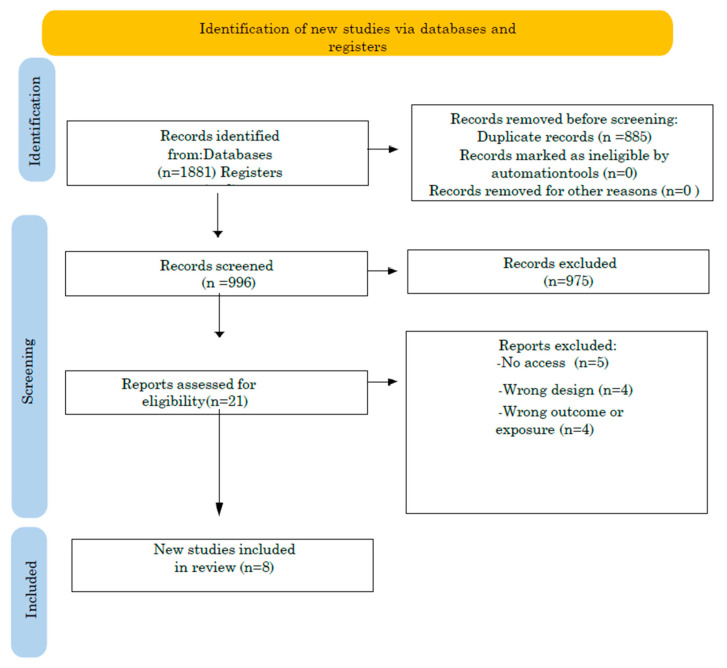
PRISMA flow diagram.

The initial database searches yielded a total of 1881 records. After the removal of 885 duplicate entries, 996 titles and abstracts were screened for relevance. Of these, 21 full-text articles were assessed for eligibility based on the predefined inclusion and exclusion criteria. Thirteen studies were excluded during the full-text review due to reasons such as insufficient methodological detail or the use of observational study designs without an intervention component. Ultimately, eight studies were included in the final analysis (see Figure 1 for the PRISMA flow diagram). The majority of the selected studies were conducted in Japan [18,19,20,21,22,23,24], with one study originating from Finland [25].

### 3.2. Characteristics of Included Studies

The eight studies included in this review involved participants who were aged 65 years or older. The follow-up periods varied significantly among the studies, ranging from as short as 2 weeks to as long as 48 months. Several outcome measures were employed to assess frailty and sarcopenia, including the Kihon Checklist (KCL), grip strength, walking speed, occlusal force, tongue pressure, and the Barthel Index. The interventions administered across these studies primarily included oral rehabilitation, functional training, swallowing therapies, and preventive oral healthcare programs.

One study by Tuuliainen et al. [25] focused on the differences in oral hygiene habits between frail and non-frail individuals. The study found that frail participants exhibited significantly lower rates of tooth brushing and denture cleaning compared to their non-frail counterparts. Although preventive interventions in this study were effective in reducing plaque levels, they did not lead to significant improvements in frailty scores. In contrast, Shiraishi et al. [18] evaluated a multidisciplinary oral care program that combined professional cleaning, patient education, and routine maintenance. This intervention not only improved oral health markers but was also positively associated with improved functional recovery and dysphagia outcomes in post-stroke patients, as evidenced by significant improvements in functional independence and food intake (*p* < 0.01).

### 3.3. Impact of Dental Interventions on Sarcopenia and Frailty

Table 1 provides an overview of the included studies, detailing the study population, intervention characteristics, frailty and sarcopenia measures, and the main reported outcomes.

#### 3.3.1. Oral Hygiene and Preventive Care

The review revealed that preventive oral hygiene interventions have the potential to improve oral health, yet their impact on frailty indices remains modest. For instance, Tuuliainen et al. [25] demonstrated that while improvements in plaque reduction were observed following preventive interventions, there was no significant change in frailty scores. Conversely, Shiraishi et al. [18] reported that a comprehensive, multidisciplinary oral care program resulted in improved oral health outcomes that were indirectly linked to better functional recovery in stroke rehabilitation patients. The improvement in dysphagia and food intake suggests that enhanced oral hygiene may have secondary benefits that contribute to overall frailty-related outcomes.

#### 3.3.2. Oral Exercises and Functional Training

The effectiveness of oral exercises in mitigating frailty and sarcopenia was evaluated in two studies. Shirobe et al. [19] implemented an intervention focused on oral exercises aimed at improving muscle strength and weight gain. The study reported significant improvements in muscle strength along with a reduction in frailty scores by an average of 1.1 points (95% CI: −1.5 to −0.7, *p* < 0.01). In another study, Kito et al. [20] found that interventions designed to enhance masticatory efficiency through structured oral exercises resulted in a moderate reduction in frailty scores (−0.6 points, 95% CI: −1.0 to −0.2, *p* < 0.05). These findings suggest that targeted oral exercises may enhance neuromuscular coordination, leading to improvements in systemic muscle function and thereby reducing the progression of frailty.

#### 3.3.3. Swallowing and Multidisciplinary Therapy

Comprehensive interventions that integrated oral exercises with physical training and nutritional guidance were examined in two studies. Ono et al. [21] and Iwao et al. [22] investigated the effects of multidisciplinary therapy programs that combined oral health interventions with additional components such as swallowing therapy and physical rehabilitation. Both studies reported significant improvements in grip strength, with increases of 1.8 kg (*p* = 0.03) and 1.5 kg (*p* = 0.003), respectively, and enhancements in walking speed (approximately +0.2 m/s, *p* = 0.04). Notably, these combined interventions were associated with significant reductions in frailty indices (approximately −0.8 points with a 95% CI of −1.2 to −0.4, *p* = 0.01). The results indicate that integrating oral health interventions within a broader framework that includes nutritional and physical support can have synergistic effects, leading to improved functional outcomes beyond what is achievable with isolated interventions.

#### 3.3.4. Long-Term Effects of Oral Health and Nutrition Education

Two studies evaluated the long-term effects of interventions that combined oral health and nutrition education. Tomata et al. [23] implemented an oral health and nutrition (OHN) education program over a period of 28 months and found that the intervention group exhibited a significantly lower incidence of disability compared to controls (*p* = 0.018). These findings suggest that sustained oral health interventions, when combined with nutritional guidance, may contribute to long-term frailty prevention. In another study, Izumi et al. [24] focused on the impact of tongue-cleaning interventions on respiratory function. Although the study did not directly assess frailty outcomes, it reported significant increases in peak expiratory flow rate (PEFR; *p* < 0.01), indicating that improved respiratory function through targeted oral interventions may indirectly support overall physical performance in older adults.

### 3.4. Certainty of Evidence

The certainty of evidence for the various outcomes was assessed using the GRADEpro software. Most studies were rated as low or very low due to limitations such as small sample sizes, lack of blinding, and variability in the frailty assessment tools used. However, the evidence supporting the impact of oral exercises on frailty indicators, as reported by Shirobe et al. [19] and Kito et al. [20], was considered to be of moderate certainty. This suggests that while there is promising evidence for the efficacy of targeted oral exercises, further research using well-controlled, larger-scale studies with standardized outcome measures is necessary to confirm these findings.

## 4. Discussion

The findings of this systematic review provide critical insight into the role of oral health interventions in mitigating frailty and sarcopenia in older adults. The evidence suggests that interventions targeting oral function—including preventive oral hygiene, structured oral exercises, and swallowing therapies—can lead to measurable improvements in frailty- and sarcopenia-related outcomes. However, significant limitations in study design, sample size, and methodological consistency limit the strength of these conclusions and underscore the need for further research.

Preventive oral hygiene interventions were found to improve oral function, as evidenced by better oral hygiene practices and reduced plaque levels [16,17]. Nevertheless, these improvements did not translate into substantial reductions in frailty scores. This observation implies that while restoring oral health is an essential component of overall well-being, it may be insufficient on its own to counteract the multifactorial nature of systemic frailty and sarcopenia. The association between increased biting force and reduced frailty risk [18] highlights the necessity of preserving masticatory function as part of a comprehensive geriatric care strategy. Future studies should explore whether additional interventions, such as dietary modifications and systemic exercise programs, could further enhance the benefits of prosthetic rehabilitation and other oral health interventions.

Oral exercises have shown a more pronounced effect on frailty indicators. Studies by Shirobe et al. [19] and Kito et al. [20] provide evidence that structured oral exercises can lead to significant improvements in muscle strength and body weight, along with meaningful reductions in frailty scores. These findings align with existing research suggesting that oral functional training can enhance neuromuscular coordination and have systemic benefits. Nonetheless, a critical gap remains in understanding the optimal frequency, intensity, and long-term sustainability of these exercises. Future research should aim to refine these exercise protocols and assess their integration with broader frailty management programs to optimize outcomes.

Swallowing therapies, particularly when combined with physical training and nutritional guidance, have also shown promising effects on functional outcomes such as grip strength and walking speed [21,22]. These multidisciplinary interventions suggest that a comprehensive approach addressing multiple aspects of physical function can yield better results than isolated oral health interventions. The significant improvements observed in grip strength and walking speed indicate that these interventions may provide systemic benefits that extend beyond improvements in oral function alone. However, given the limited number of studies in this area, additional research is needed to establish standardized protocols and to assess the long-term benefits of such interventions in diverse settings.

Long-term interventions that integrate oral health with nutrition education appear to offer sustained benefits. The study by Tomata et al. [23] demonstrated that an oral health and nutrition education program could reduce the incidence of disability over a 28-month period. This finding suggests that ongoing, sustained interventions are essential for achieving lasting improvements in functional independence among older adults. While Izumi et al. [24] focused on tongue-cleaning interventions and reported significant improvements in respiratory function, the indirect benefits of such interventions on frailty warrant further investigation. Improvements in respiratory function, as indicated by increased peak expiratory flow rate, may contribute to overall physical performance and should be considered as part of a comprehensive approach to frailty prevention.

The certainty of evidence across the included studies is a concern, largely due to the methodological limitations that were common among them. Many studies were limited by small sample sizes, lack of blinding, and the use of heterogeneous outcome measures. Despite these challenges, the moderate-certainty evidence supporting the efficacy of oral exercises provides a promising avenue for further exploration. It is imperative that future studies employ larger, randomized controlled trials with standardized assessment tools to confirm the preliminary findings reported in this review. In addition, research should explore the underlying biological mechanisms that link improved oral health with enhanced systemic function. Investigations into inflammatory biomarkers, muscle metabolism, and the neuromuscular effects of oral exercises could provide valuable insights into how these interventions exert their benefits. Emerging evidence suggests that low-grade chronic inflammation—reflected by markers such as IL-6 and CRP—may contribute to the link between oral health and systemic conditions like frailty and sarcopenia [26,27]. While a detailed discussion of inflammatory pathways remains outside the primary scope of this review, this represents an intriguing field for future research that may yield valuable insights into potential therapeutic strategies.

The clinical implications of these findings are significant. Integrating oral health interventions into routine geriatric care could serve as an effective strategy for preventing or delaying the progression of frailty and sarcopenia. Early identification of oral frailty by dental professionals may allow for timely interventions that can mitigate broader systemic decline. The incorporation of oral health evaluations into comprehensive geriatric assessments is an approach that holds promise for improving overall patient outcomes. Moreover, fostering interdisciplinary collaborations between dental professionals, geriatricians, nutritionists, and physical therapists is crucial for developing holistic care plans tailored to the unique needs of older adults.

Despite the promising trends observed, future research must address several key areas to strengthen the evidence base. Larger-scale studies with robust methodological designs are needed to confirm the benefits of oral health interventions. Standardization of outcome measures will be essential to facilitate comparisons across studies and to build a coherent body of evidence. In addition, research into the cost-effectiveness of these interventions is needed to inform policy decisions and ensure that effective strategies are accessible to the broader aging population. By addressing these gaps, future studies can contribute to the development of more effective and sustainable approaches to managing frailty and sarcopenia.

## 5. Conclusions

Oral health interventions, including preventive care, structured oral exercises, and swallowing therapies, have the potential to positively influence outcomes related to frailty and sarcopenia in older adults. The studies reviewed in this systematic analysis suggest that improvements in oral function are associated with enhancements in grip strength, walking speed, and reductions in frailty indices, particularly when these interventions are implemented as part of a multidisciplinary care strategy. Despite the methodological variability, small sample sizes, and inconsistencies in outcome measures across studies, the overall evidence supports the integration of oral health management into routine geriatric care.

Future research should prioritize well-designed randomized controlled trials that employ standardized frailty and sarcopenia measures to better delineate the impact of oral health interventions. Long-term studies are also necessary to determine the sustainability of these interventions and to explore their role in preventing frailty-related disability over time. Moreover, establishing interdisciplinary collaborations between dental and geriatric professionals will be essential for developing comprehensive care strategies that address the multifactorial nature of aging-related decline. Ultimately, the integration of oral health interventions into broader healthcare practices may play a critical role in enhancing the quality of life and functional independence of older adults.

## Figures and Tables

**Table 1:** Evidence synthesis.

Study (Author, Year)	Participants (% Female)	Age (Years)	Setting/Length of Follow-Up	Fragility Measure	Intervention	Overview Results	Overview Impact on Fragility/Sarcopenia	Certainty of Evidence
Tuuliainen et al. (2020) [25]	231 (75%)	>75	Community-dwelling/6 months	Abbreviated CGA (cognitive, functional, and depression scales)	Denture hygiene education, oral mucosa cleaning, and nutritional guidance	Increased denture cleaning frequency (mean: +1.4 times/day, *p* < 0.05), reduced plaque (*p* < 0.01)	No significant frailty change: frailty score mean −0.3 (95% CI: −0.6 to 0.0)	Low
Shirobe et al. (2022) [19]	219 (75%)	Not reported	Community-dwelling/8 weeks	Tanaka Method: frailty assessment based on weight, BMI, grip strength, walking speed, and KCL	Tongue pressure training, masticatory exercises, and prosodic training	Significant increase in body weight (+1.2 kg, *p* < 0.05), BMI (+0.5, *p* = 0.002), grip strength (+1.5 kg, *p* < 0.01)	Reduction in frailty index: mean −1.1 (95% CI: −1.5 to −0.7, *p* < 0.01)	Moderate
Kito et al. (2019) [20]	86 (93%)	>65	Residential care/12 weeks	Occlusal force, tongue pressure, tongue–lip motor function, grip strength, Timed Up and Go test	Oral exercises combined with dietary modifications (textured foods, chewing training)	Improved masticatory efficiency: particle size −1.5 mm (*p* < 0.05)	Moderate frailty reduction: frailty index change −0.6 (95% CI: −1.0 to −0.2, *p* < 0.05)	Moderate
Iwao et al. (2019) [22]	43 (84%)	≥65	Residential care/3 months	KCL, grip strength (<26 kg men/<18 kg women), timed up-and-go (>12 s)	Combined oral health and physical training (chewing exercises and balance tasks)	Improved grip strength (+2.4 kg, *p* < 0.05), reduced bacterial count (*p* < 0.01)	KCL frailty score improvement: −1.2 points (95% CI: −1.8 to −0.6, *p* = 0.002)	Very Low
Ono et al. (2017) [21]	Residents in LTCF	Not reported	Residential care/3 months	Kihon Checklist (KCL), grip strength, walking speed	Long-term care prevention program (oral care, supervised swallowing training)	Improved grip strength (+1.8 kg, *p* = 0.03), increased walking speed (+0.2 m/s, *p* = 0.04)	Reduction in frailty index: −0.8 points (95% CI: −1.2 to −0.4, *p* = 0.01)	Very Low
Shiraishi et al. (2021) [20]	300 (78%)	72 (mean)	Hospitalized/48 months	ROAG (voice, lips, mucous membranes, tongue, gums, teeth, saliva, swallowing)	Oral screening, assessment, education, counseling, oral and dysphagia rehabilitation, and medical treatment by dentists	Better oral health was associated with improved functional recovery and swallowing function	Improved food intake level scale (FILS) (*p* < 0.01) and modified Rankin Scale (mRS) (*p* < 0.001)	Very Low
Izumi et al. (2019) [24]	114 (75%)	≥65	Residential care/2, 4, and 12 weeks	Barthel Index, Peak Expiratory Flow Rate (PEFR)	Tongue-cleaning intervention	Increased PEFR significantly (*p* < 0.01), more effective in low BI group	Improvement in respiratory function but no direct frailty assessment	Low
Tomata et al. (2017) [23]	192 (64 intervention, 128 control) (66%)	≥65	Mixed (includes hospitalized, residential care and community-dwelling)/28 months	KCL	Complex educational program: oral health and nutrition (OHN)	There was a lower incidence of disability in the intervention group compared to the control (*p* = 0.018)	Significant reduction in disability-related frailty outcomes (HR = 0.78, *p* < 0.05)	Very Low

## Data Availability

The search files (.ris format) used and/or analyzed during the current study are available from the corresponding author upon reasonable request.

## Abbreviations

The following abbreviations are used in this manuscript:

PRISMA	Preferred Reporting Items for Systematic Reviews and Meta-Analyses
MeSHs	Medical Subject Headings
KCL	Kihon Checklist
CGA	Cognitive, functional, and depression scales
BMI	Body mass index
PEFR	Peak expiratory flow rate
OHN	Complex educational program: oral health and nutrition
GRADEpro	Guideline Development Tool

## Appendix A

**Table A1:** Search strategies.

Database	Search Query
PubMed	(“Dental Care”[MeSH Terms] OR “Oral Health”[MeSH Terms] OR “Mouth Rehabilitation”[MeSH Terms] OR “Dentures”[MeSH Terms]) AND (“Frailty”[MeSH Terms] OR “Physical Frailty”[MeSH Terms] OR “Frail Elderly”[MeSH Terms]) OR (“Sarcopenia”[MeSH Terms] OR “Muscle Weakness”[MeSH Terms])
Scopus	TITLE-ABS-KEY(“Dental Care” OR “Oral Health” OR “Mouth Rehabilitation” OR “Dentures”) AND TITLE-ABS-KEY(“Frailty” OR “Physical Frailty” OR “Frail Elderly”) OR TITLE-ABS-KEY(“Sarcopenia” OR “Muscle Weakness” OR “Muscle Strength”)
SciELO	(“Cuidado Dental” OR “Salud Bucal” OR “Rehabilitación Oral” OR “Prótesis Dentales”) AND (“Fragilidad” OR “Fragilidad Física” OR “Anciano Frágil”) OR (“Sarcopenia” OR “Debilidad Muscular” OR “Fuerza Muscular”)
